# Regenerative medicine curriculum for next-generation physicians

**DOI:** 10.1038/s41536-019-0065-8

**Published:** 2019-02-07

**Authors:** Saranya P. Wyles, Richard E. Hayden, Fredric B. Meyer, Andre Terzic

**Affiliations:** 1 Department of Dermatology, Rochester, MN USA; 2 Mayo Clinic Center for Regenerative Medicine, Rochester, MN USA; 3 Department of Otolaryngology, Phoenix, AZ USA; 4 Mayo Clinic Alix School of Medicine, Rochester, MN USA; 5 Department of Neurologic Surgery, Rochester, MN USA; 6 Department of Cardiovascular Medicine, Rochester, MN USA; 7 Department of Molecular Pharmacology and Experimental Therapeutics, Rochester, MN USA; 8 Department of Clinical Genomics, Mayo Clinic, Rochester, MN USA

## Abstract

Regenerative sciences are poised to transform clinical practice. The quest for regenerative solutions has, however, exposed a major gap in current healthcare education. A call for evidence-based adoption has underscored the necessity to establish rigorous regenerative medicine educational programs early in training. Here, we present a patient-centric regenerative medicine curriculum embedded into medical school core learning. Launched as a dedicated portal of new knowledge, learner proficiency was instilled by means of a discovery–translation–application blueprint. Using the “from the patient to the patient” paradigm, student experience recognized unmet patient needs, evolving regenerative technologies, and ensuing patient management solutions. Targeted on the deployment of a regenerative model of care, complementary subject matter included ethics, regulatory affairs, quality control, supply chain, and biobusiness. Completion of learning objectives was monitored by online tests, group teaching, simulated clinical examinations along with longitudinal continuity across medical school training and residency. Success was documented by increased awareness and proficiency in domain-relevant content, as well as specialty identification through practice exposure, research engagement, clinical acumen, and education-driven practice advancement. Early incorporation into mainstream medical education offers a tool to train next-generation healthcare providers equipped to adopt and deliver validated regenerative medicine solutions.

## Clinical practice in the regenerative medicine era

Regenerative technologies, aimed at restoring form and function, inform the prospect of transforming standard-of-care practices.^1,2^ The evolution from the traditional perspective of “fighting disease” to the increasingly actionable paradigm of “restoring health” begets a new skillset imperative for the developing healthcare practitioner.^3^ To ensure that regulated regenerative therapies are provided for patient care, educating a specialized workforce that can distinguish safe and valid regenerative options is warranted.^4,5^ Regenerative approaches, however, remain underemphasized in medical school education, including in the United States.^6,7^ As a result, there is a paucity of physicians adequately trained in regenerative principles and practices, necessitating earlier and systematic introduction to this transdisciplinary field that imposes a novel lexicon and new know-how.^8,9^

A national effort aims to bridge curricular gaps. These include the Wake Forest Regenerative Medicine Essentials Course, University of Pittsburgh McGowan Institute Regenerative Medicine Summer School, Harvard Stem Cell Institute Medical Scientist Training Fellowship, Duke Scholars Program in Oncology and Regenerative Medicine, Education and Training at Institute for Stem Cell Biology & Regenerative Medicine Stanford School of Medicine that expose physician-scientists to the regenerative vocabulary and core principles. Similar international efforts are recognizing a diverse regenerative armamentarium. Case in point, stem cell-based therapies are the mainstay of regenerative biotherapies today, yet a spectrum of tissue-engineering and acellular/molecular regenerative approaches are increasingly assessed.^10,11^ Accordingly, new and complementary skillsets will be needed to prepare physicians in order to meet future demand in regenerative practice.^12^ Indeed, prevailing healthcare epidemics imposed by age-related degenerative conditions, namely cardiovascular disease, cancer, and/or diabetes,^13^ mandate timely regenerative education.^14^

The landmark 21st Century Cures Act provided support from US Congress for personalized medicine approaches including regenerative medicine.^15^ Beyond ensuring product quality, safety and efficacy, the Food and Drug Administration (FDA) has underscored the need to accelerate new therapies enabled by regenerative sciences.^4,16^ As the clinical landscape is reshaped by regenerative technologies, there is a pressing call for resources and training for primary care doctors and general practitioners, along with specialists, who are involved in long-term patient care.^8,17^ Developing genuine proficiency in regenerative medicine at the medical school level is essential as healthcare adapts to impending delivery requirements.^18^

## Preparing the next-generation physician

As a multidisciplinary integrated model in addressing patient needs, Mayo Clinic has recognized regenerative medicine as a strategic investment in the future of healthcare. Institutional strategy, envisioned as a science-driven practice advancement priority, is executed through a discovery–translation–application mandate, deployed across medical, surgical, radiological, and laboratory medicine specialties.^1,19^

### Curriculum development

Integral to the regenerative medicine roll-out is the build-out of a specialized workforce equipped with skills to carry-out regenerative care. The regenerative medicine curriculum for medical students offers a comprehensive educational experience that encompasses discovery, development, and delivery of next-generation patient management modalities targeted to address root cause of disease.^20^ Guiding principles for the introductory “Regenerative Medicine and Surgery Course” included: (i) early introduction of regenerative medicine concepts in medical education training; (ii) dynamic teaching methods such as interactive, simulation, and laboratory experiences to maximize student engagement; (iii) multidisciplinary, patient-centric approach to comprehend bench-to-bedside translation and iterative optimization; (iv) all-inclusive group discussion involving patients and faculty along with students; and (v) online education modules and medical student presentations to ensure learning proficiency. Encompassing a patient-centric paradigm, the “Regenerative Medicine and Surgery Course” is a prototype dedicated to medical students and integrated within the medical school curriculum.

### Discovery–translation–application scope and content

**Fig. 1:**
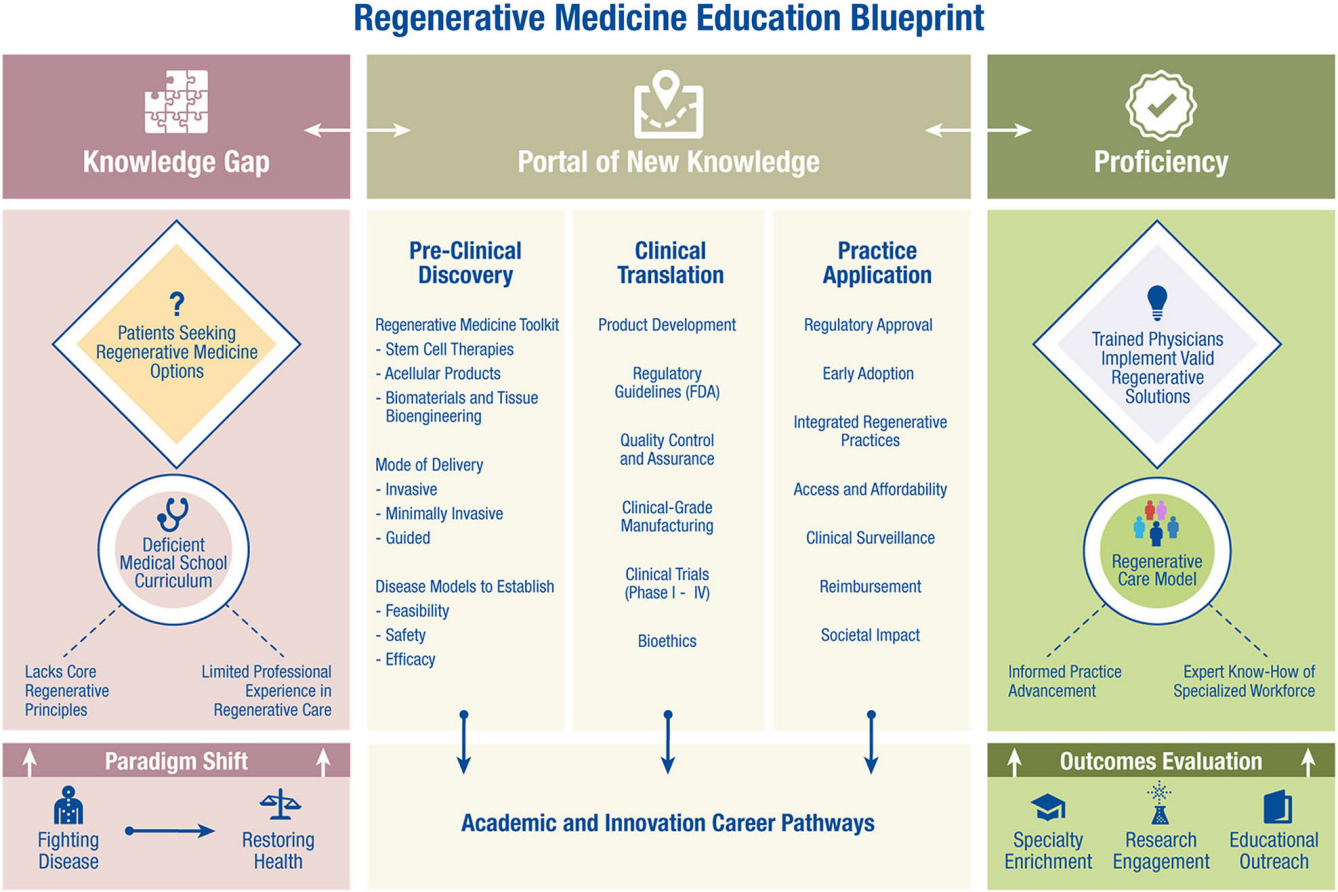
Regenerative medicine education blueprint. Current knowledge gap in medical school curriculum is evident in the setting of increasing patient inquiries about regenerative medicine options. Given the paradigm shift in healthcare, from the traditional perspective of “fighting disease” to the increasingly actionable paradigm of “restoring health”, the developing physician needs to enter the portal of new knowledge for a skillset encompassing preclinical discovery, clinical translation, and practice application. Through this education blueprint, the next-generation healthcare provider can be trained to implement valid regenerative solutions and advance the regenerative care model

The “Regenerative Medicine and Surgery Course” spans comprehension of regenerative technologies translated across relevant medical and surgical specialties. Education modules include regenerative medicine principles, bench-to-bedside translation, clinical-grade biomanufacturing and regulatory science, regenerative procedures, and integration of regenerative practices in patient care (Fig. 1). Student participation is tailored toward an understanding of regenerative medicine objectives;^21^ recognition of patient options offered by regenerative therapies; ability to describe diagnostic and therapeutic applications including implementation of regenerative medicine workups; and engagement with the patient population seeking regenerative solutions.

Multifaceted content is exemplified by sessions on clinically applied concepts including bone marrow transplantation for hematological malignancies; nerve reconstruction in neurosurgery; facial reanimation and composite allotransplant in plastic and microsurgery; hybrid core decompression and osteochondral grafts in orthopedics; platelet-rich plasma interventions in physical and sports medicine; cell therapies for neurodegenerative, cardiac, and kidney disease; prenatal fetoscopic regenerative interventions; and neorganogenic regeneration for aerodigestive pathologies.^22–25^ The range of presented clinical trials underscores the ongoing assessment and validation stage of emerging therapies, i.e., mesenchymal stem cells in amyotrophic lateral sclerosis and multiple systems atrophy; mononuclear cell therapy for hypoplastic left heart syndrome; lineage-specified cardiopoietic stem cells for chronic heart failure; stem cells for bronchiolitis obliterans, renal artery stenosis, avascular necrosis of the hip, and osteoarthritis of the knee; stem cell-coated fistula plugs for Crohn’s disease; stromal cells for host versus graft disease; and stem cells for relapsed ovarian cancer.^1,26–30^

Adoption of knowledge through hands-on regenerative procedures guides student understanding. The bench-to-bedside translational curriculum highlights the importance of preclinical models for testing feasibility, safety and efficacy,^31^ and teaches students how regenerative interventions are utilized in clinical trials along with ethical issues that have influenced regenerative research and adoption in practice.^32^ As medical students progress through the curriculum, opportunities to understand and demonstrate proficiency in a patient consult service are presented. Furthermore, exposure to Current Good Manufacturing Practice (cGMP) allows students to learn how standardized and scalable regenerative products are generated compliant with regulations for clinical-grade manufacturing, quality control/assurance, and delivery.^33–35^ Other regenerative technologies and resources included exposure to the bio-insurance principle realized in the biobanking/biotrust platform, product/process development encompassing tissue engineering and (a)cellular regenerative biotherapies, and innovator/entrepreneurial pathway. At the course conclusion, next-generation physicians learned how to incorporate regenerative strategies into future clinical training and understand the steps of discovering, perfecting, and building through scale-in/scale-out paradigms.^36^

### Curriculum implementation

The inaugural “Regenerative Medicine and Surgery Course” introduced regenerative medicine concepts early in medical education training, and while launched at Mayo Clinic was made accessible to national and international audience of trainees. Participants included medical students in preclinical (first and second year) and clinical years (fourth year) in addition to graduate students (Ph.D. and M.D., Ph.D. trainees), internal medicine residents (PGY-1 and PGY-2) and research fellows. Dedicated elective and research time allowed participation during full-time curriculum. The multidisciplinary approach continues to serve as a facilitator, incorporating interactive and hands-on experiences, to better engage students. A team of student champions were assembled to map curriculum content, timeline of execution and resource acquisition. Beyond didactic lectures, students engage in a daily clinical trial highlight from surgical and medical specialties that focuses on how regenerative approaches are optimized in the clinic. Laboratory experiences include exposure to novel technologies such as stem cell culture, disease models, and 3D bioprinting. Medical students learn translation from disease models pertaining to stem cell delivery and tissue engineering. Hands-on surgical procedures demonstrate, in the anatomy cadaver laboratory, ultrasound-guided joint injection and surgical procedures for degenerative conditions. Subject matter experts in medical and surgical specialties including cardiology, otolaryngology, orthopedic surgery, plastic surgery, and sports medicine lead these laboratory demonstrations, highlighting the biology of regenerative medicine concomitantly with reconstructive procedures. To foster student–faculty mentorship, a regenerative medicine career panel offers prospects in innovative translational research and transformative clinical practice. Intramural synergy between Mayo Clinic Center for Regenerative Medicine and Mayo Clinic School of Medicine enabled financial support for faculty time, laboratory resources, anatomy dissection, patient–actor simulation center, and internal facility resources. Extramural funding secured the opportunity for external participants.

The evolving nature of regenerative sciences mandate that each course iteration incorporates previous experiences while presenting the latest trends and clinical experiences. The course plasticity allows for medical students to participate in concept-based and practice-applied sciences across the progressive discovery–translation–application continuum.

### Outcomes

Completion of learning objectives is monitored by online tests, group teaching, simulated clinical examinations along with continuity across medical school training. Success is documented by increased awareness and proficiency in domain-relevant content, as well as specialty identification through practice exposure, research engagement, clinical acumen, and education-driven practice advancement.

Over the 5-year developmental period of this course, the curriculum met its objectives to increase student literacy in regenerative medicine and inspired a sizeable percentage of participants to pursue expanded degree programs in this area. Specifically, target metrics were to increase medical student knowledge of regenerative medicine (measured by pretest and posttest learning objectives; students achieved over 50% improvement), to build interest in regenerative solutions for clinical application (measured by specialty identification and student research engagement), and to engage education-driven practice advancement (measured by pursuit of additional degrees/research fellowships in regenerative sciences laboratories). While the course was developed for medical students, it has birthed a new cadre of investigators proficient in regenerative sciences through extended training in Master’s or Ph.D. programs.

**Fig. 2:**
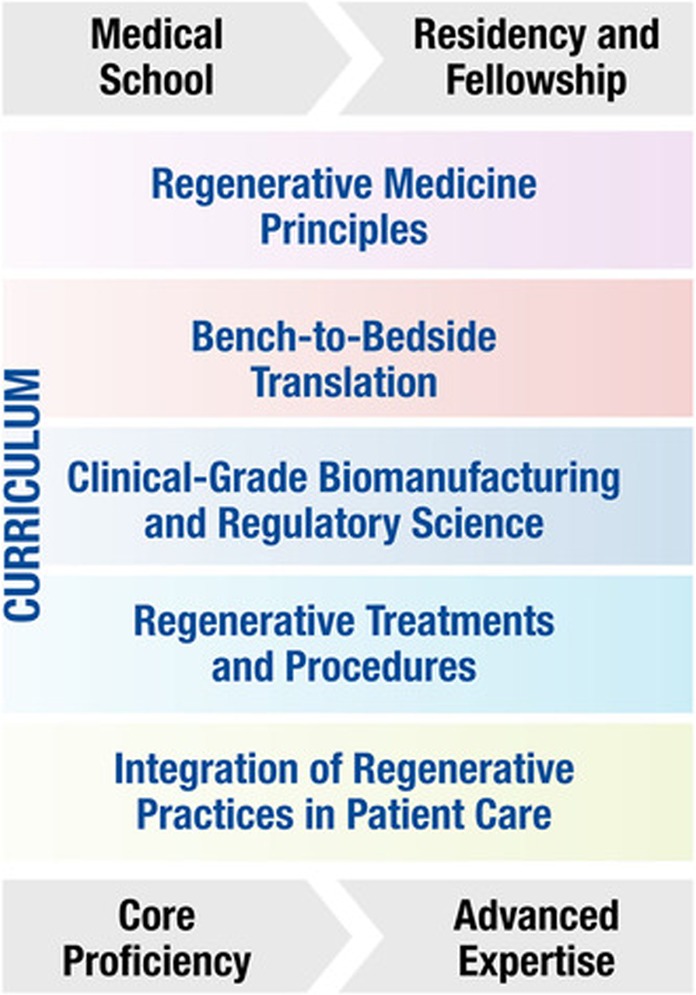
Regenerative medicine and surgery curriculum. Fundamental principles of the “regenerative medicine and surgery course” curriculum are introduced early in medical school training, and longitudinally expanded in residency and clinical fellowship, allowing for core proficiency to develop into advanced expertize of the next-generation specialized workforce

Integration of regenerative medicine across medical school training continues to be an important goal to retain/expand physician–investigators in this field. To this end, acquired knowledge early in medical school training is re-enforced at Mayo Clinic in later medical school years (through a dedicated “Clinical Regenerative Medicine Elective”), and longitudinally expanded in residency and clinical fellowship (Fig. 2). Advanced education contributes to a specialized workforce ready to practice informed regenerative care. In this way, core proficiency acquired early can be propagated and fully developed into expert know-how over the continuum of medical training.

## Regenerative medicine shapes education

Regenerative therapies will permeate the future clinical landscape, in particular for diseases that have been proven intractable to current management strategies.^37^ Yet, education in regenerative medicine is lagging behind scientific and clinical advances. This threatens to leave the physicians-in-training ill-equipped to address the changing needs in patient care.^38^ A systematic review of medical school curricula included no reports of regenerative medicine courses dedicated for medical students.^39^ In line with the projection that regenerative care will represent 10% of all healthcare in the next decade,^40^ a comprehensive, patient-centered course is needed to prepare healthcare providers.

Here, we present an innovative curriculum that addresses this recognized knowledge gap by educating next-generation learners. As such, this transdisciplinary training is a prototype that can promote education-driven practice advancement and could serve as a playbook to be implemented globally. The course offered an unprecedented opportunity to enrich the medical school curriculum with disruptive innovation. Long-term follow-up is needed to determine the efficacy of such educational experiences in developing the next-generation workforce in the practice of regenerative sciences and care. Beyond physicians-in-training, the shifting composition of the healthcare workforce^41^ will require training opportunities inclusive of the evolving landscape of advanced practitioners.

## Data Availability

Data sharing not applicable to this article as no datasets were generated or analyzed during the current study.
